# Supplementary material for: Two C-terminal sequence variations determine differential neurotoxicity between human and mouse α-synuclein
Source: Mol Neurodegener. 2020 Sep 8;15:49. doi: 10.1186/s13024-020-00380-w (PMC7487555; doi:10.1186/s13024-020-00380-w)
Supplement: Supplementary file 1 — Additional file 1: Figure S1. Differences in antibody recognition of the different aSyn variants. Related to Fig. 3. Figure S2. Human/mouse mismatches at positions 121 and 122 do not affect the ability of aSyn to form Proteinase K-resistant aggregates in rat striatum. Related to Fig. 4. Figure S3. Effects of human/mouse substitutions at position 87 on fibrillization rates of aSyn variants. Related to Fig. 5. Table S1. Membrane affinities and maximum helix content of aSyn variants titrated with PG:PC SUVs. Table S2. P values for vesicle permeabilization data in Fig. 7C. [file 13024_2020_380_MOESM1_ESM.pdf]

Supplementary Information

SI Figure Legends

Figure S1. Differences in antibody recognition of the different aSyn variants. Related to Figure 3. Adjacent rat midbrain sections of high-titer groups were stained with human aSyn-specific syn211 antibody (A-E) or for comparison with pan-aSyn-specific Syn-1 antibody (F-J). Corresponding contralateral (intact, uninjected) sides are shown in panels A and F, respectively. Scale bar, 50 µm.

Figure S2. Human/mouse mismatches at positions 121 and 122 do not affect the ability of aSyn to form Proteinase K-resistant aggregates in rat striatum. Related to Figure 4. Striatal specimens of animals from the highest vector group were incubated in the presence of Proteinase K for 0 min (B-E), 5 min (G-J), or 45 min (L-O) and stained for aSyn to reveal the formation of digestion-resistant aggregates (arrows). The digestion of endogenous (and therefore soluble) aSyn was monitored in the CA2/CA3 region of the hippocampus (A, F, K). Scale bar, 25 µm.

Figure S3. Effects of human/mouse substitutions at position 87 on fibrillization rates of aSyn variants. Related to Figure 5. The formation of amyloid-like fibrils was monitored in solutions of h-aSyn A53T and h-aSyn A53T/S87N (A), h-aSyn Chimera and h-aSyn Chimera S87N (B), m-aSyn and m-aSyn N87S (C), or m-aSyn Chimera and m-aSyn Chimera N87S (D) (35 µM of each). The protein solutions were incubated at 37°C with constant agitation and analyzed at various times for thioflavin T fluorescence. The graphs show the mean normalized fluorescence (determined from 3 or 4 technical replicates in each experiment) plotted against the incubation time.

SI Tables

Table S1. Membrane affinities and maximum helix content of aSyn variants titrated with PG:PC SUVs[a]

Variant	K_d[b] (μM)	ellipticity minimum (x 10^3 deg·cm^2/dmol)	maximum helicity (%)
h-aSyn-WT	1.5 ± 0.4	-18.6 ± 0.8	50 ± 3
h-aSyn-A53T	2.5 ± 0.3	-20.5 ± 0.4	55 ± 2
m-aSyn	2.4 ± 0.2	-20.4 ± 0.4	55 ± 2
h-aSyn-Chimera	2.6 ± 0.2	-21.1 ± 0.4	56 ± 2
m-aSyn-Chimera	1.6 ± 0.2	-17.7 ± 0.3	47 ± 2

[a]Values (± standard error) were determined from the far-UV CD data in Fig. 7A using equations 3-6; the concentration of aSyn was 5 μM.

[b]The CD data obtained for each variant were fit to equation 3 with the *N* value (binding stoichiometry) set to 160, the value obtained by fitting the h-aSyn-WT data to equation 3 without any constraints on *N*.

Table S2. *P* values for vesicle permeabilization data in Figure 7C[a]

Comparison	Time (h)				
	24	48	72	96	120
ctrl vs. h-aSyn	****	****	****	****	****
ctrl vs. h-aSyn A53T	****	****	****	****	****
ctrl vs. m-aSyn Chimera	****	****	****	****	****
ctrl vs. h-aSyn Chimera	****	****	****	****	****
ctrl vs. m-syn	****	****	****	****	****
h-aSyn vs. h-aSyn A53T	ns	ns	ns	ns	ns
h-aSyn vs. m-aSyn Chimera	ns	ns	ns	ns	ns
h-aSyn vs. h-aSyn Chimera	ns	ns	****	****	****
h-aSyn vs. m-aSyn	ns	ns	**	**	****
h-aSyn A53T vs. m-aSyn Chimera	ns	ns	ns	ns	ns
h-aSyn A53T vs. h-aSyn Chimera	ns	***	****	****	****
h-aSyn A53T vs. m-aSyn	ns	**	**	*	****
m-aSyn Chimera vs. h-aSyn Chimera	ns	*	***	****	****
m-aSyn Chimera vs. m-aSyn	ns	ns	ns	ns	***
h-aSyn Chimera vs. m-aSyn	ns	ns	ns	***	*

[a]Two-way ANOVA; *p<0.05, **p<0.01, ***p<0.001, ****p<0.0001.

Figure S1.

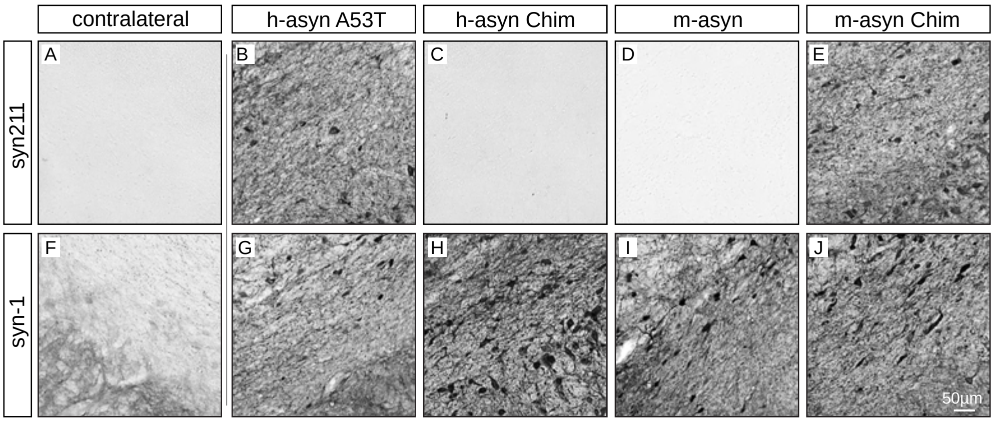

contralateral
h-asyn A53T
h-asyn Chim
m-asyn
m-asyn Chim
syn211
syn-1
A
B
C
D
E
F
G
H
I
J
50µm

Figure S2

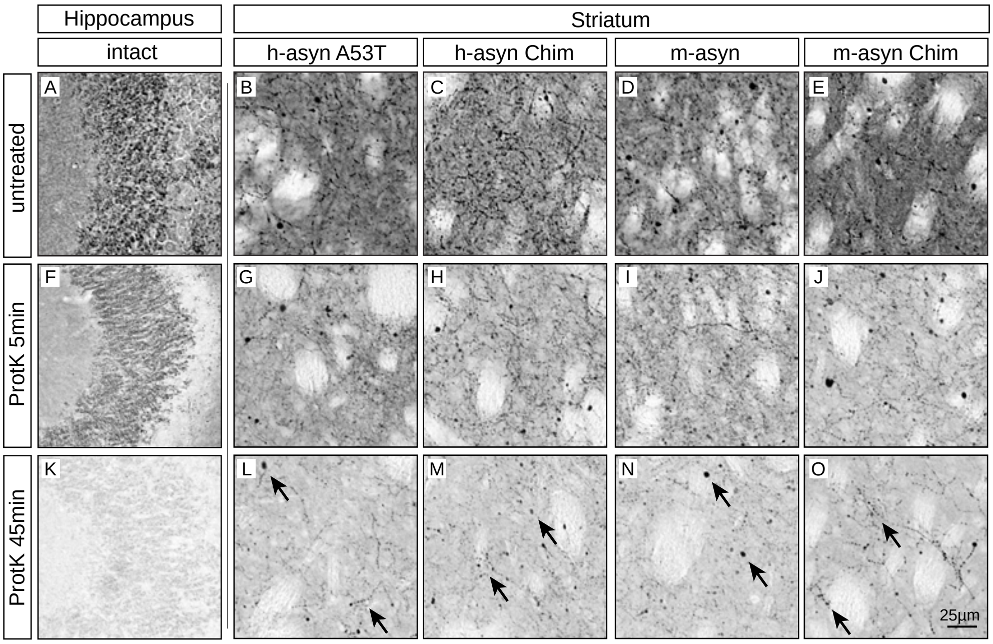

Hippocampus
Striatum
intact
h-asyn A53T
h-asyn Chim
m-asyn
m-asyn Chim
untreated
ProtK 5min
ProtK 45min
A
B
C
D
E
F
G
H
I
J
K
L
M
N
O
25µm

Figure S3

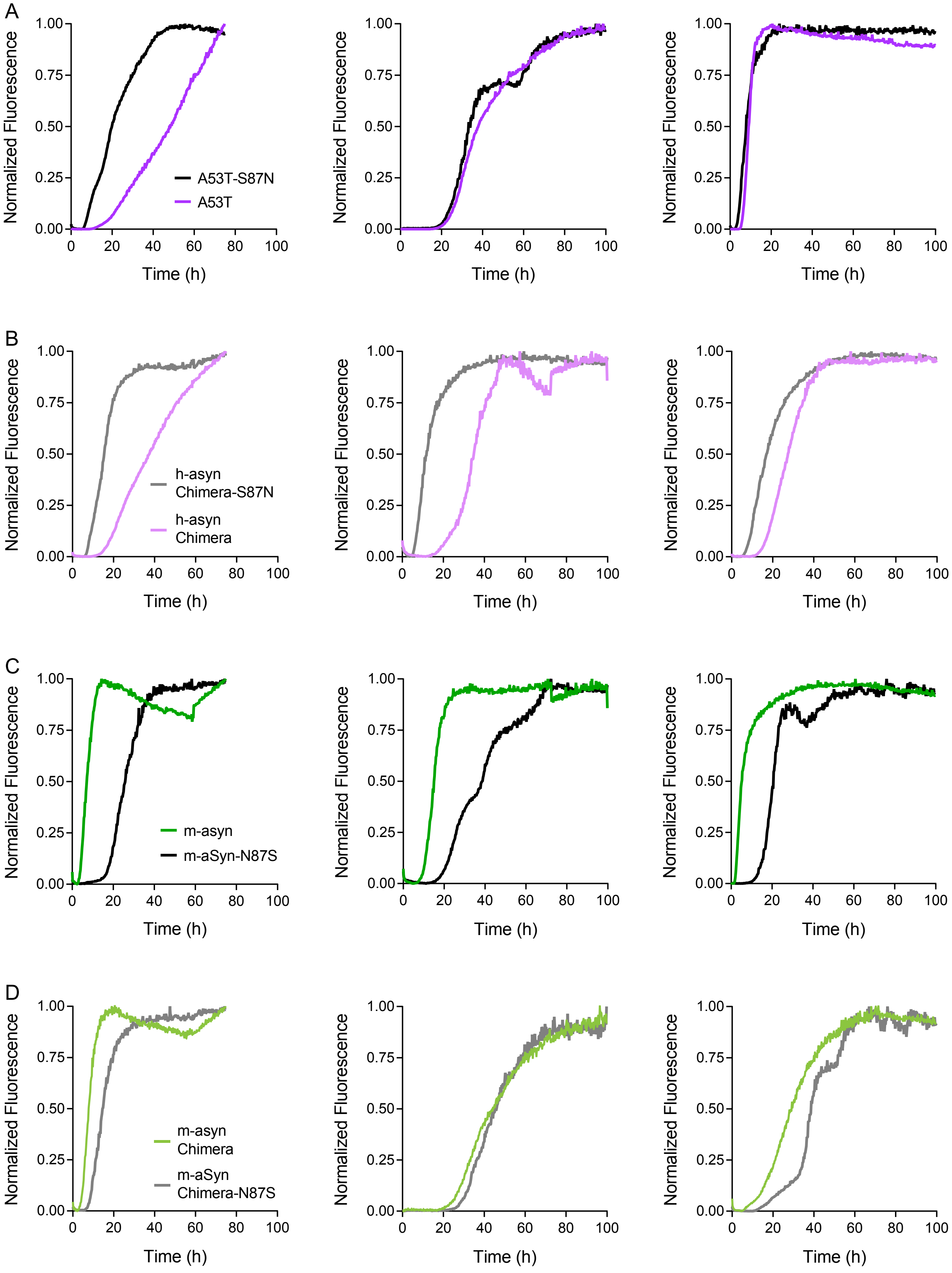
A
Normalized Fluorescence
Time (h)
A53T-S87N
A53T
B
h-asyn
Chimera-S87N
h-asyn
Chimera
C
m-asyn
m-aSyn-N87S
D
m-asyn
Chimera
m-aSyn
Chimera-N87S
